# An algorithm for the classification of study designs to assess diagnostic, prognostic and predictive test accuracy in systematic reviews

**DOI:** 10.1186/s13643-019-1131-4

**Published:** 2019-09-03

**Authors:** Tim Mathes, Dawid Pieper

**Affiliations:** Institute for Research in Operative Medicine, Witten/Herdecke University, Ostmerheimer Str. 200, 51109 Cologne, Germany

**Keywords:** Study design classification, Diagnostic accuracy, Sensitivity, Specificity, Diagnosis prognosis, Prediction

## Abstract

Results of medical tests are the main source to inform clinical decision making. The main information to assess the usefulness of medical tests for correct discrimination of patients are accuracy measures. For the estimation of test accuracy measures, many different study designs can be used. The study design is related to the clinical question to be answered (diagnosis, prognosis, prediction), determines the accuracy measures that can be calculated and it might have an influence on risk of bias. Therefore, a clear and consistent distinction of the different study designs in systematic reviews on test accuracy studies is very important. In this paper, we propose an algorithm for the classification of study designs of test accuracy, that compare the results of an index test (the test to be evaluated) with the results of a reference test (the test whose results are considered as correct/the gold standard) studies in systematic reviews.

## Background

Results of medical tests are the main source to inform clinical decision making. Test accuracy is the ability of a test to discriminate between different patient groups (e.g. healthy and diseased). The first step in assessing the value of a medical test before performing comparative impact studies (e.g. randomised controlled trials) on different tests is the assessment of the test accuracy. Moreover, if impact studies are absent, evidence on test accuracy can be used to estimate effects on patient important outcomes by linking the evidence of the different care pathways (e.g. no treatment vs. treatment) resulting from the different test-based classifications to the test accuracy measures (e.g. false negative test results) [1].

The use of test, even the same test in health care can be manifold regarding the clinical question (e.g. diagnosis of a health status, prediction of therapy success) and purpose (e.g. screening or surveillance, treatment monitoring or staging). Moreover, medical tests are usually not used standing alone but in different constellations with other tests, including triage before another test, add-on to another test and parallel testing with another test.

In addition to the manifold application areas, test accuracy studies are often unclearly labelled in the medical literature regarding the differentiation between diagnosis, prognosis and prediction (for example, see [2–8]), and regarding the underlying epidemiological study design (for example, see [8–15]). These aspects complicate the correct classification of the study design.

Systematic reviews on test accuracy (e.g. on sensitivity and specificity) summarise test accuracy measures from several studies. A consistent and clear definition of the study designs is critical for the quality at several tasks of the systematic review. This includes selection of studies, choosing the tool for risk of bias assessment, deciding which studies should be pooled in the same meta-analysis and assessing the certainty of the body of evidence [16].

In the following, we propose an algorithm for the classification of test accuracy studies in systematic reviews.

### Preliminary considerations

This algorithm only applies to studies comparing the results of an index test (the test to be evaluated) with the results of a reference test (the test whose results are considered as correct/the gold standard). The tests of interest must allow a binary classification, either by using a cut-off for a categorical or continuous measure (e.g. high vs. low blood pressure, score of a prognostic model) or be binary in nature. The algorithm can be used for any test used in health care. This test can be a single test (e.g. imaging) or a predefined combination (AND or OR link) of tests (e.g. imaging and laboratory) or factors (e.g. symptoms, patient characteristics) that are formally combined in a diagnostic or prognostic model [17, 18]. When reviewers apply the algorithm, they should be aware that the test must not be a test in narrow sense (e.g. laboratory tests, diagnostic devices). It can also be an observation (e.g. healthy), medical procedure (e.g. general health check) or clinical assessment (e.g. inspection of the corpse).

The algorithm cannot be used for studies on test calibration and studies on test reliability (e.g. test-retest studies). The algorithm can further not be used for classifying comparative and impact studies on tests. These are all studies that compare accuracy of at least to tests using the same reference standard or studies that compare the impact of different tests on health outcomes (e.g. a randomised controlled trial of that compares two different screening strategies regarding the impact on mortality) [19, 20]. However, it is important to regard that in comparative studies on tests, single arms of the study in which a test is performed can be considered as test accuracy studies (e.g. the arm of a randomised controlled trial in that a screening test is used) and thus might be (potentially) relevant for the systematic reviews on test accuracy. Studies in which a relative effect measures is calculated but no test accuracy measure can be calculated (e.g. prognostic factor studies) are also not considered in this paper because this can be classified as studies on exposures (e.g. case-control studies) [21]. For this studies on exposures as well as comparative impact studies, classifications have been described elsewhere [19, 22, 23].

### The classification algorithm

**Fig. 1:**
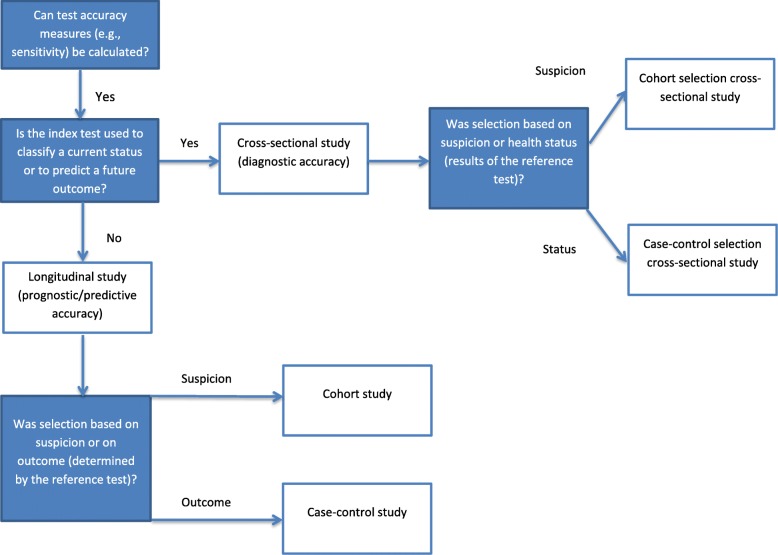
Algorithm for classification of test accuracy study desings

The classification algorithm is presented in Fig. 1. The study designs that can be classified with the algorithm are shown in Table 2. In the following paragraphs, the application of the algorithm is explained. For illustration, the reader might imagine a systematic review on test accuracy of brief cognitive test for older people for which we provide examples throughout the description of the algorithm.

#### Is it a test accuracy study?

It is not always directly obvious if the study under consideration is indeed a test accuracy study because studies might not report accuracy measures but only provide data which enable calculating accuracy measures (e.g. sensitivity of the cognitive test for the diagnosis of dementia). In other words, systematic review authors must check if it is possible to calculate a 2 × 2 cross-tabulation (see Table 1). Therefore, the first criterion of the algorithm is the question, whether the study is a test accuracy study.

**Table 1:** 2 × 2 cross table for calculation of test accuracy measures

	Reference test	
	Positive	Negative
Index test		
Positive	True positive	False positive
Negative	False negative	True negative

#### Diagnostic, prognostic or predictive test accuracy (cross-sectional or longitudinal)?

Tests in healthcare can be used for diagnosis, prognosis and/or prediction. Diagnosis refers to the “probability that a specific outcome or disease is present (or absent) within an individual, at *this point in time*” [24]. This means, in diagnostic accuracy studies, the test information is used to make a classification of a current health status (e.g. cognitive impaired vs. healthy). In contrast, “prognosis refers to the risk of (any) *future* health outcomes in people with a given disease or health condition” (e.g. high risk vs. low risk to die within 1 year) [25]. For tests, this means prognostic and predictive accuracy studies classify the risk for developing an outcome in the future, which is not present at the time the test is applied. Prognosis can be further subdivided in prognostic and predictive research. Prognosis considers the natural course of diseases and thus answers the question who needs treatment (e.g. there is only a need for treatment if there is a risk for developing dementia). Prediction aims to predict the outcome in treated patients and thus answers the question who and how should be treated (e.g. cognition training in people with mild cognitive impairment is only necessary if there is a chance of improvement) [24]. In the following, we will consider prognosis and prediction tests together because both have a longitudinal view from present in the future and therefore their test accuracy can be assessed with the same study designs. Nevertheless, systematic review authors should carefully consider if the study under assessment considers the natural course of diseases (prognosis) or considers treated patients (prediction).

The second criterion of the classification algorithm is the question if the objective of the study under consideration is to assess the diagnostic accuracy or the prognostic/predictive accuracy of a test. As the main difference between these two is the time component (current vs. future status), the second classification criterion considers the time interval between index and reference test. A diagnosis is the classification of a current status. All information on an *individual* participant refers to the same time-point (e.g. cognitive test indicates that the patient currently has dementia). This implies that all diagnostic accuracy studies are cross-sectional in nature [26, 27]. Because a diagnosis provides information on a current status, the reference test and the index test should be performed at the same time-point. When applying this criterion, it is important to refer it to the time-point of collecting information on index and reference test for an *individual* study participant and not to the time-point of data collection for the study (e.g. chart review to verify the diagnosis of dementia) to avoid confusion. For example, a patient might receive an index test (e.g. brief cognitive test) in primary care and the reference test (e.g. comprehensive cognitive assessment) at a hospital stay several months later. The information on both test results is collected from routinely collected health care data at the same time point (e.g. a patient registry of geriatric patients). Although the data for the study are collected at the same time-point from the registry, the study is not cross-sectional because index and reference test are not performed at the same time at the *individual* participant level. In practice, the time-points at which the tests are performed are usually not exactly the same. Thus, the same time-point can mean almost at the same time-point (e.g. brief cognitive test and comprehensive cognitive assessment at the same visit) or that one test is performed nearby the other (e.g. brief cognitive test and comprehensive cognitive assessment at the same hospital stay). One should judge if the time interval in the study under assessment was adequate, considering the probability that the patient’s status (e.g. no cognitive impairment) has not changed between the index and the reference test [28]. Consequently, the acceptable delay depends on the condition and is larger in slowly progressing conditions than in fast progressing conditions. For the study design classification, this means, if it can be justified that is improbable that the status has changed (e.g. diagnosis of Alzheimer dementia), studies with a delay between index and reference test might also be classified as cross-sectional. As it cannot be excluded that the patient’s status has changed between the two tests, there is a risk of misclassification bias in diagnostic accuracy studies because the ratio of patient groups (e.g. proportion classified as cognitive impaired or not cognitive impaired) resulting from the test classification might have changed in the meantime [29]. We suggest that two time-intervals between index and reference test are pre-specified in systematic reviews on diagnostic test accuracy. One for the decision about inclusion in the systematic review and another criterion (usually a smaller time interval) for judging low/moderate risk of delayed verification bias [29]. The specification of the thresholds would usually require the expertise of a methodologist and a clinician.

A prognosis/prediction is a classification of a future status. In studies on prognosis/prediction, the index test is used to classify participants according to their risk for developing a certain outcome (e.g. progression of mild cognitive impairment to dementia), or therapy response (e.g. a response to cognitive training). Here, the reference test is used to assess the outcome status. The information of index and reference test results for an *individual* participant refer to different time-points. This implies that studies on prognosis/prediction are always longitudinal because there are repeated observations, namely the result of the index test and later on the results of the reference test for each participant [24]. In contrast to diagnostic accuracy studies, the time interval between index test and reference test should not be too short but “sufficiently” long. The time interval should be chosen in such a way that, if the outcome of interest has not occurred (e.g. negative test for dementia), it is improbable that it will occur soon thereafter (e.g. the mild cognitive impairment will probably not progress to dementia in the next months). In addition to a lifetime period, often information on certain pre-defined time intervals is clinical relevant (e.g. developing dementia in the next 5 years). However, in research practice, the choice of the time interval can be driven rather by the availability of data (e.g. length of follow-up) than by clinical importance. In addition to the judgement of clinical relevance, the time interval in the study under consideration is critical for the risk of bias assessment. An insufficient length of follow-up can cause lead-time bias in studies with unblinded index test results because in participants with a positive index test (e.g. indication for cognitive impairment), the occurrence of an event is suspected (e.g. developing dementia). Therefore, participants with a positive index test often have a higher chance to be monitored more closely, and consequently also have a higher chance for receiving the reference test earlier (e.g. through more intensive monitoring of cognitive function) than participants with a negative index test result. Moreover, the observation of fewer events in one group can be spurious if the test result is only associated with a delay of events but actually not lowers the event rate considering a lifetime period. Therefore, as for diagnosis, we suggest that systematic review authors pre-specify two time intervals. One for selecting studies that should be chosen depending on the time horizon of interest (e.g. early or late progression) and one for judging the studies’ risk of bias [30]. It is important to note that to our knowledge for studies on prognostic accuracy, no tool for assessing the methodological quality exists.

Systematic review authors will regularly be interested in either diagnostic accuracy (e.g. diagnosis of mild cognitive impairment) or prognostic/predictive accuracy (e.g. predicting dementia in patients with mild cognitive impairment). A pre-specification of the time intervals for selecting studies is therefore very important to distinct diagnostic from prognostic/predictive studies, in particular, because the same test can often be used for diagnosis as well as prognosis/prediction (see for example [31, 32]). This means that the clinical question cannot be always deduced from the test itself but that only the time interval between the index test and reference test indicates if the study is on concurrent or predictive accuracy. Moreover, the distinction might be difficult because the passage from delayed verification to prognosis/prediction can be fluent.

If systematic review authors are convinced that the test can exclusively be used for either diagnosis or prognosis/prediction, they can use only the respective (diagnosis, prognosis/prediction) path of the algorithm.

#### Cohort type or case-control selection of participants?

The second criterion distinguishes cohort type studies from case-control type studies and can be applied for diagnostic accuracy studies in a similar way than for prognostic/predictive accuracy studies.

In general, cohort type studies and case-control type studies are distinguished by the method of selecting the participants for the study [33, 34]. In cohort type test accuracy studies, the participants are recruited based on suspicion. By suspicion we mean, that there is an indication to perform the test, including signs and symptoms, the presence of risk factors (e.g. patient characteristics, environment) or results of previous medical tests.

Theoretically, in population screening, people might be selected regardless whether there is an indication to do so or not. However, in practice, this is not the usual case, but also in most population-based screening programs, there is at least a vague indication to perform a test (e.g. certain age group, gender). In cohort designs, all suspicious participants receive the index test and the reference test to determine their current status (diagnosis) or to assess their outcome status (prognosis/prediction). In diagnostic cohort type studies, the index test and the reference test are performed at the same time. This cross-sectional relationship implies that the order of the reference and the index test can differ as long as the tests are performed at (almost) the same time or without too much delay (see above). Thus, the reference and the index test can be performed simultaneously, the reference test can be performed after the index test or the index test can be performed after the reference test. For cohort type studies on prognostic/predictive accuracy, the longitudinal relationship implies that the index test is always performed before the reference test.

In case-control designs, the selection of participants is based on the health status/outcome. The results of the index test of participants with a positive reference test result/event (cases) are compared to the results of the index test of participants with a negative reference test result/no event (controls). Similar to case-control studies on exposures or interventions, cases and controls might come from the same source (e.g. a registry) or different sources (e.g. cases from an Alzheimer registry and controls form an administrative database). In case-control diagnostic accuracy studies, the reference test on the *individual* participant level is always performed before the index test but the view/interpretation (e.g. retrospective record review) on the results of the index test is always retrospective. It is important to note that in case-control designs, no predictive values can be calculated because the prevalence/incidence (column sum in the 2 × 2 table of participants classified positive and negative with the reference test) is an artificial result of the design (e.g. 50% in 1:1 case-control matching).

We suggest labelling diagnostic accuracy studies with patient selection based on suspicion “cohort selected cross-sectional studies” and studies with case-based sampling “case-control selected cross-sectional studies”. This labelling ensures a clear differentiation to longitudinal study designs and indicates the participant selection method. Although, we are aware that combining the labels cohort and cross-sectional virtually appears to be contrary, we believe that labelling it like this is preferable to a completely new labelling because most reviewers are familiar with these standard selection methods.

Figure a and c in Table 2 illustrate the design of a “cohort selection cross-sectional study” and “case-control selection cross-sectional study”, respectively.

**Table 2:**
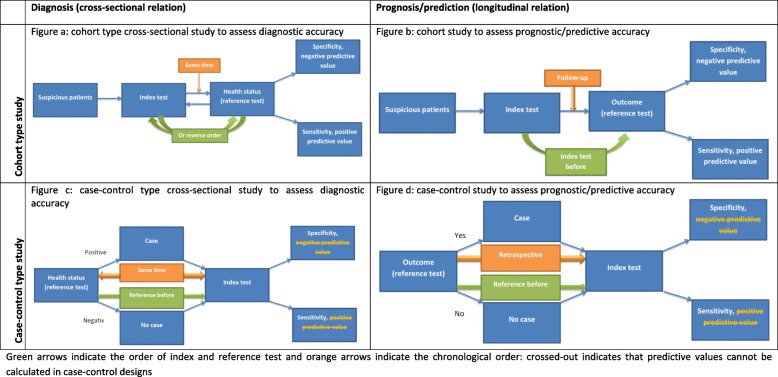
Study designs to assess test accuracy

The classifications (e.g. positive versus negative) resulting from an index test to judge prognosis/prediction can be considered as different exposures (e.g. high risk for developing dementia vs. low risk for developing dementia) and the observation period is longitudinal. The only difference to the classical cohort and case-control study in epidemiology is the effect measure (test accuracy measures instead of risk ratios). Therefore, we suggest labelling prognostic/predictive accuracy studies in the same way, namely “cohort studies” and “case-control studies”.

Figure b and d in Table 2 illustrate the design of a “cohort study” and a “case-control study”, respectively.

Either test accuracy studies might be based on data specifically collected for the study (i.e. a study database) or on already existing data sources (e.g., routinely collected data). Often the classification retrospective/prospective is used to distinct if the data were specifically collected for the study or an already existing data source was used. We recommend avoiding this classification for two reasons. Firstly, often studies have prospective (e.g. analysis plan) as well as retrospective aspects (e.g. data collection) [35]. Secondly, especially for diagnostic accuracy studies, this would lead to cumbersome classifications (e.g. retrospective cross-sectional study). Instead, the data source used for the study should be clearly described in the systematic review.

#### Illustrating examples

Table 3 shows an illustrating example for each test accuracy study type. In example study 1 [36], all kidney transplant recipients of at least 50 years received a faecal immunochemical test (index test) for colorectal cancer screening. Following the faecal immunochemical test, patients were referred to colonoscopy (reference test). In this study, the sampling was based on suspicion (kidney transplant recipients). The index test and the reference test were performed at the same time (disease has probably not progressed). Consequently, this study is a cohort sampling cross-sectional study on diagnostic accuracy (see Table 2 figure a).

**Table 3:** Examples for the different accuracy study types

Example study	Population	Diseases /outcome	Index test	Reference test/outcome	Time between Index and reference test	Sampling	Type
1 [30]	Kidney transplant recipients aged ≥ 50 years	Colorectal caner	Faecal immunochemical testing	Colonoscopy with histological evaluation	Directly referred to reference after receiving the index test	All kidney transplant recipients aged ≥ 50 years	Diagnostic accuracy cohort-type cross-sectional study
2 [31]	Attended a memory clinic	Alzheimer dementia	Cognitive test	Clinical diagnosis of Alzheimer’s	Reference test and index test during outpatient visit	Three age matched controls were selected for each patient with Alzheimer’s disease	Diagnostic accuracy case-control type cross-sectional study
3 [32]	Age 50–90 years	Osteoporotic fractures	Prognostic model	Hip fracture	5 years	All patients in a payer provider health organisation	Prognostic accuracy cohort study
4 [33]	Men ≥ 40 years	Prostate cancer	Prostate specific antigen screening using blood draws	Prostate cancer diagnosed using biopsy	Mean 7.1 years	540 cases and 1034 controls matched for age and date of blood draw	Prognostic accuracy case-control study

In the second example [37], patients with a clinical diagnosis (reference test) of Alzheimer (cases) attended a memory clinic were matched to participants without Alzheimer, who were recruited from relatives accompanying patients to the memory clinic (no disease, controls). Patients as well as relatives received a cognitive test (index test) during the visit at the memory clinic. The participant sampling was based on disease in one group and absence of diseases in the other. Although, the reference test was performed at another time as the index test, it can be considered as the same time-point because the disease could not have been resolved, i.e. is still a current status. Consequently, this study is a case-control sampling cross-sectional diagnostic accuracy study (see Table 2 figure c).

The third example [38] examines all patients between 50 and 90 years (suspicion) in a payer provider health organisation. In the study, patient characteristics and other factors were formally combined in a prognostic model. The prognostic model calculates a score that is dichotomised using different cut-offs (index test). For each participant, the risk for developing fractures within 5 years (future event) was predicted. Sampling was based on suspicion and a future outcome was predicted. Although it is not fully clear from the publication, it can be assumed that most patients were not treated for osteoporosis. Consequently, the study is a cohort study to assess prognostic accuracy (see Table 2 figure b).

The last example study [39] included men of at least 40 years (suspicion), who had results of a blood draw from a larger population-based cohort study. Patients with prostate cancer (outcome event) were sampled and matched to patients without prostate cancer (no outcome event, controls). The prostate-specific antigen levels (index test) of the prior blood draw were categorised and compared. Participants were untreated, sampling was based on outcome and a future outcome is predicted. Consequently, the study is a (nested) case-control study to assess prognostic accuracy (see Table 2 figure d).

### Limitations

Our algorithm only covers the basic design features of test accuracy studies. Further criteria exist that are important for the risk of bias assessment and for assessment of confidence in the body of evidence. In particular, the sampling method is important in this respect. Cohort type studies with a consecutive or random sample (e.g. one arm of a randomised controlled trail) are considered to provide least biased information on test accuracy. In addition, the study population should be representative for the target population so that externally valid accuracy measures can be obtained [27, 29, 33, 40].

## Conclusion

We suggest an algorithm for the classification of test accuracy studies in systematic reviews. We hope that it will facilitate and improve consistent classification of test accuracy studies in systematic reviews. Future studies should test the practicability and reliability of the classification algorithm.

## Data Availability

Not applicable.

## References

[1] Schünemann HJ, Mustafa R, Brozek J, Santesso N, Alonso-Coello P, Guyatt G, Scholten R, Langendam M, Leeflang MM, Akl EA (2016). GRADE Guidelines: 16. GRADE evidence to decision frameworks for tests in clinical practice and public health. J Clin Epidemiol.

[2] Bae JH, Park SH, Ye BD, Kim SO, Cho YK, Youn EJ, Lee HS, Hwang SW, Yang DH, Kim KJ (2017). Development and validation of a novel prediction model for differential diagnosis between Crohn's disease and intestinal tuberculosis. Inflamm Bowel Dis.

[3] Hilvering B, Vijverberg SJH, Jansen J, Houben L, Schweizer RC, Go S, Xue L, Pavord ID, Lammers JJ, Koenderman L (2017). Diagnosing eosinophilic asthma using a multivariate prediction model based on blood granulocyte responsiveness. Allergy.

[4] Giannini V, Mazzetti S, Marmo A, Montemurro F, Regge D, Martincich L (2017). A computer-aided diagnosis (CAD) scheme for pretreatment prediction of pathological response to neoadjuvant therapy using dynamic contrast-enhanced MRI texture features. Br J Radiol.

[5] Dubey D, Singh J, Britton JW, Pittock SJ, Flanagan EP, Lennon VA, Tillema JM, Wirrell E, Shin C, So E (2017). Predictive models in the diagnosis and treatment of autoimmune epilepsy. Epilepsia.

[6] Chitty LS, Finning K, Wade A, Soothill P, Martin B, Oxenford K, Daniels G, Massey E (2014). Diagnostic accuracy of routine antenatal determination of fetal RHD status across gestation: population based cohort study. BMJ (Clin Res Ed).

[7] Nwachuku EL, Balzer JR, Yabes JG, Habeych ME, Crammond DJ, Thirumala PD (2015). Diagnostic value of somatosensory evoked potential changes during carotid endarterectomy: a systematic review and meta-analysis. JAMA Neurol.

[8] van den Bosch WB, Mangnus L, Reijnierse M, Huizinga TW, van der Helm-van Mil AH (2015). The diagnostic accuracy of the squeeze test to identify arthritis: a cross-sectional cohort study. Ann Rheum Dis.

[9] Andreeva E, Pokhaznikova M, Lebedev A, Moiseeva I, Kozlov A, Kuznetsova O, Degryse JM (2015). The RESPECT study: RESearch on the PrEvalence and the diagnosis of COPD and its tobacco-related etiology: a study protocol. BMC Public Health.

[10] Perry JJ, Stiell IG, Sivilotti ML, Bullard MJ, Emond M, Symington C, Sutherland J, Worster A, Hohl C, Lee JS (2011). Sensitivity of computed tomography performed within six hours of onset of headache for diagnosis of subarachnoid haemorrhage: prospective cohort study. BMJ (Clin Res Ed).

[11] Nickolas TL, O'Rourke MJ, Yang J, Sise ME, Canetta PA, Barasch N, Buchen C, Khan F, Mori K, Giglio J (2008). Sensitivity and specificity of a single emergency department measurement of urinary neutrophil gelatinase-associated lipocalin for diagnosing acute kidney injury. Ann Intern Med.

[12] Craig JC, Williams GJ, Jones M, Codarini M, Macaskill P, Hayen A, Irwig L, Fitzgerald DA, Isaacs D, McCaskill M (2010). The accuracy of clinical symptoms and signs for the diagnosis of serious bacterial infection in young febrile children: prospective cohort study of 15 781 febrile illnesses. BMJ (Clin Res Ed).

[13] Luqmani R, Lee E, Singh S, Gillett M, Schmidt WA, Bradburn M, Dasgupta B, Diamantopoulos AP, Forrester-Barker W, Hamilton W (2016). The role of ultrasound compared to biopsy of temporal arteries in the diagnosis and treatment of giant cell arteritis (TABUL): a diagnostic accuracy and cost-effectiveness study. Health Technol Assess (Winchester, England).

[14] Acute Abdominal Pain (AAP) Study group (2016). Diagnostic accuracy of surgeons and trainees in assessment of patients with acute abdominal pain. Br J Surg.

[15] Allen VB, Gurusamy KS, Takwoingi Y, Kalia A, Davidson BR (2016). Diagnostic accuracy of laparoscopy following computed tomography (CT) scanning for assessing the resectability with curative intent in pancreatic and periampullary cancer. Cochrane Database Syst Rev.

[16] Hartling L, Bond K, Santaguida PL, Viswanathan M, Dryden DM (2011). Testing a tool for the classification of study designs in systematic reviews of interventions and exposures showed moderate reliability and low accuracy. J Clin Epidemiol.

[17] Steyerberg EW, Moons KGM, van der Windt DA, Hayden JA, Perel P, Schroter S, Riley RD, Hemingway H (2013). Altman DG, for the PG: prognosis research strategy (PROGRESS) 3: prognostic model research. PLoS Med.

[18] van Stralen KJ, Stel VS, Reitsma JB, Dekker FW, Zoccali C, Jager KJ (2009). Diagnostic methods I: sensitivity, specificity, and other measures of accuracy. Kidney Int.

[19] Mustafa RA, Wiercioch W, Cheung A, Prediger B, Brozek J, Bossuyt P, Garg AX, Lelgemann M, Büehler D, Schünemann HJ (2017). Decision-making about healthcare related tests and diagnostic strategies: a review of methodological and practical challenges. J Clin Epidemiol.

[20] Leeflang MMG, Reitsma JB. Systematic reviews and meta-analyses addressing comparative test accuracy questions. Diagn Progn Res. 2018;2(1):17. 10.1186/s41512-018-0039-0. 10.1186/s41512-018-0039-0 PMC6460833 31093565

[21] Riley RD, Hayden JA, Steyerberg EW, Moons KGM, Abrams K, Kyzas PA, Malats N, Briggs A, Schroter S, Altman DG (2013). Prognosis research strategy (PROGRESS) 2: prognostic factor research. PLoS Med.

[22] Mathes T, Pieper D (2017). Study design classification of registry-based studies in systematic reviews. J Clin Epidemiol.

[23] Ferrante di Ruffano L, Hyde CJ, McCaffery KJ, Bossuyt PMM, Deeks JJ (2012). Assessing the value of diagnostic tests: a framework for designing and evaluating trials. BMJ (Clin Res Ed).

[24] Collins GS, Reitsma JB, Altman DG, Moons KG (2015). Transparent reporting of a multivariable prediction model for individual prognosis or diagnosis (TRIPOD): the TRIPOD statement. BMC Med.

[25] Hemingway H, Croft P, Perel P, Hayden JA, Abrams K, Timmis A, Briggs A, Udumyan R, Moons KGM, Steyerberg EW (2013). Prognosis research strategy (PROGRESS) 1: a framework for researching clinical outcomes. BMJ.

[26] Knottnerus JA, Muris JW (2003). Assessment of the accuracy of diagnostic tests: the cross-sectional study. J Clin Epidemiol.

[27] Bossuyt PM (2008). LMCDCfISIC, September HfSRoDTAVu.

[28] Kamarudin AN, Cox T, Kolamunnage-Dona R (2017). Time-dependent ROC curve analysis in medical research: current methods and applications. BMC Med Res Methodol.

[29] Whiting PF, Rutjes AW, Westwood ME, Mallett S, Deeks JJ, Reitsma JB, Leeflang MM, Sterne JA, Bossuyt PM (2011). QUADAS-2: a revised tool for the quality assessment of diagnostic accuracy studies. Ann Intern Med.

[30] Hayden JA, van der Windt DA, Cartwright JL, Cote P, Bombardier C (2013). Assessing bias in studies of prognostic factors. Ann Intern Med.

[31] Creavin ST, Wisniewski S, Noel-Storr AH, Trevelyan CM, Hampton T, Rayment D, Thom VM, Nash KJ, Elhamoui H, Milligan R, et al. Mini-mental state examination (MMSE) for the detection of dementia in clinically unevaluated people aged 65 and over in community and primary care populations. Cochrane Database Syst Rev. 2016;(1):CD011145. 10.1002/14651858.CD011145.pub2 PMC8812342 26760674

[32] Arevalo-Rodriguez I, Smailagic N, IFM R, Ciapponi A, Sanchez-Perez E, Giannakou A, Pedraza OL, Bonfill Cosp X, Cullum S. Mini-mental state examination (MMSE) for the detection of Alzheimer’s disease and other dementias in people with mild cognitive impairment (MCI). Cochrane Database Syst Rev. 2015;(3):CD010783. 10.1002/14651858.CD010783.pub2 PMC6464748 25740785

[33] Rutjes AWS, Reitsma JB, Vandenbroucke JP, Glas AS, Bossuyt PMM (2005). Case–control and two-gate designs in diagnostic accuracy studies. Clin Chem.

[34] Mathes T, Pieper D (2017). Clarifying the distinction between case series and cohort studies in systematic reviews of comparative studies: potential impact on body of evidence and workload. BMC Med Res Methodol.

[35] Higgins JP, Ramsay C, Reeves BC, Deeks JJ, Shea B, Valentine JC, Tugwell P, Wells G (2013). Issues relating to study design and risk of bias when including non-randomized studies in systematic reviews on the effects of interventions. Res Synth Methods.

[36] Collins MG, Teo E, Cole SR, Chan C-Y, McDonald SP, Russ GR, Young GP, Bampton PA, Coates PT (2012). Screening for colorectal cancer and advanced colorectal neoplasia in kidney transplant recipients: cross sectional prevalence and diagnostic accuracy study of faecal immunochemical testing for haemoglobin and colonoscopy. BMJ.

[37] Brown J, Pengas G, Dawson K, Brown LA, Clatworthy P (2009). Self administered cognitive screening test (TYM) for detection of Alzheimer’s disease: cross sectional study. BMJ (Clin Res Ed).

[38] Dagan N, Cohen-Stavi C, Leventer-Roberts M, Balicer RD (2017). External validation and comparison of three prediction tools for risk of osteoporotic fractures using data from population based electronic health records: retrospective cohort study. BMJ (Clin Res Ed).

[39] Holmstrom B, Johansson M, Bergh A, Stenman UH, Hallmans G, Stattin P (2009). Prostate specific antigen for early detection of prostate cancer: longitudinal study. BMJ (Clin Res Ed).

[40] Schünemann HJ, Oxman AD, Brozek J, Glasziou P, Jaeschke R, Vist GE, Williams JW, Kunz R, Craig J, Montori VM (2008). Grading quality of evidence and strength of recommendations for diagnostic tests and strategies. BMJ (Clin Res Ed).

